# Niche partitioning between planktivorous fish in the pelagic Baltic Sea assessed by DNA metabarcoding, qPCR and microscopy

**DOI:** 10.1038/s41598-022-15116-7

**Published:** 2022-06-29

**Authors:** Andreas Novotny, Kinlan Mehdi Goulwen Jan, Jan Dierking, Monika Winder

**Affiliations:** 1 Department of Ecology, Environment, and Plant Sciences, Stockholm University, Stockholm, Sweden; 2 GEOMAR Helmholtz Centre for Ocean Research Kiel, Kiel, Germany; 3 Present Address: Institute for the Oceans and Fisheries, The University of British Columbia, Vancouver, Canada

**Keywords:** Ecology, Marine biology

## Abstract

Marine communities undergo rapid changes related to human-induced ecosystem pressures. The Baltic Sea pelagic food web has experienced several regime shifts during the past century, resulting in a system where competition between the dominant planktivorous mesopredatory clupeid fish species herring (*Clupea harengus*) and sprat (*Sprattus sprattus*) and the rapidly increasing stickleback (*Gasterosteus aculeatus*) population is assumed to be high. Here, we investigate diet overlap between these three planktivorous fishes in the Baltic Sea, utilizing DNA metabarcoding on the *18S rRNA* gene and the *COI* gene, targeted qPCR, and microscopy. Our results show niche differentiation between clupeids and stickleback, and highlight that rotifers play an important role in this pattern, as a resource that is not being used by the clupeids nor by other zooplankton in spring. We further show that all the diet assessment methods used in this study are consistent, but also that DNA metabarcoding describes the plankton-fish link at the highest taxonomic resolution. This study suggests that rotifers and other understudied soft-bodied prey may have an important function in the pelagic food web and that the growing population of pelagic stickleback may be supported by the open feeding niche offered by the rotifers.

## Introduction

Marine ecosystems around the globe experience rapid regime shifts induced by human activity, altering the community composition and ecosystem functions^1^. While some species are pushed to the edge of extinction, new opportunities open for those that can adapt to the new conditions and fill open niches that the regime shift brings with it. To monitor ecosystem resilience and understand why some populations decline while others thrive, a detailed understanding of the food web composition and structure is required^2^. Despite this, the basic trophic interactions remain poorly resolved in most marine food web studies. Planktivorous fish are often clustered in the same feeding niche, even though they and their prey (e.g., zooplankton) span a broad diversity of sizes, taxa, and ecological functions^3,4^. By contributing substantially to the pelagic biomass, small pelagic fish occupy a key function connecting lower and upper trophic levels^5,6^. Thus, variation in zooplankton availability directly affects the growth and survival of these mesopredators, and ultimately the success of fish recruitment, while the top-down mechanisms induced by small pelagic fish on zooplankton indirectly control phytoplankton^7^.

The Baltic Sea is strongly influenced by human stressors^8^, and consequently has undergone a sequence of ecosystem-wide regime shifts during the last century^9^. A dramatic decline of the main pelagic predator, the piscivorous Atlantic cod (*Gadus morhua*) around the year 1990 created the pelagic ecosystem regime that we, to a large extent, still experience today^7,10^, dominated by the clupeid mesopredators European sprat (*Sprattus sprattus*, hereafter ‘sprat’) and Atlantic herring (*Clupea harengus*, hereafter ‘herring’). Density-dependent competition for food resources has resulted in reduced stomach fullness and body mass of the two clupeids in the Baltic Sea proper^11^. However, a large partition of the pelagic zooplankton production remains unutilized by the clupeids, leaving an open niche that could potentially be occupied^12^. In parallel to the increase in clupeid abundance, research highlighted an exponential increase in another pelagic mesopredator, the three-spined stickleback (*Gasterosteus aculeatus,* hereafter ‘stickleback’) over the last two decades^13,14^ that caused a trophic cascade in coastal spawning areas, down to the algal level^15,16^. Although the dramatic increase of stickleback was triggered by alterations in the dominance of predators and prey in coastal areas^17^, the open pelagic habitat, where stickleback are assumed to complete a substantial part of their life cycle^13,17^, also needs to offer enough resources to support the growing population.

Most studies of fish stomach content are based on microscopic observations, where identification relies on residuals of undigested body parts, often hard exoskeletons. As a result, copepods and cladocerans are described being the main food for planktivorous fish, leading to a high diet overlap between sprat, herring, and stickleback in the Baltic Sea^12^. At the same time, soft and gelatinous zooplankton (i.e., rotifers and ctenophores) can at times dominate the zooplankton biomass in the Baltic Sea^18–20^. However, the digestion rate for soft and gelatinous prey is faster compared to crustaceans^21^, and potentially contribute to an overestimation of reported empty fish gut content. DNA metabarcoding has proven to be a successful tool investigating diverse groups of diets of zooplankton, being able to detect a broad diversity of prey items for zooplankton in the pelagic ecosystem^20,22^. A recent study using DNA metabarcoding conducted in coastal spawning areas was able to detect a much broader diversity of stickleback prey preference than previously described, indicating flexibility and adaptivity of the stickleback^23^. However, the same method has not yet been used to study diet overlap between the three pelagic mesopredators in the open pelagic water.

In this study, we assess diet overlap between the three most abundant planktivorous mesopredators in the Baltic Sea, sprat, herring, and stickleback. We utilized traditional microscopic analysis, DNA metabarcoding and quantitative PCR (qPCR) to reveal prey items that are difficult to assess microscopically. Here we ask if soft-bodied plankton, such as rotifers and ctenophores, are important prey items of planktivorous fishes and whether clupeids and stickleback compete for the same resources.

## Results

The metabarcoding of adult fish gut contents produced 3.3 and 2.9 million *18S rRNA* (*18S*) and *COI* gene sequences, respectively, that passed quality control, of which 0.5 and 0.1 million of the sequences were identified as fish (belonging to family Teleostei; *18S*) and host (*COI*), respectively, and were excluded in all downstream analyses (Table 1). Sprat larval stages produced 0.6 million *18S* and 0.9 million *COI* reads, of which 20,264 and 61,409 reads were kept after filtration, respectively (Table 1).

**Table 1:** Summary of the sample size (n), sequence reads (million) and unique amplicons variants (ASVs) for the *18S* and *COI* barcodes associated to all adult fish and larvae species and individual adult and larvae species at each step of the downstream data processing. The first filtration was performed to remove host sequences and the rarefaction was performed for adult life stages based on the sample with lowest read count (see "Methods").

Gene region	Life stage	Fish species	Before filtration			After filtration			After rarefaction
			n	ASV	Reads (M)	n	ASV	Reads (M)	ASV
*18S*	Adult	Herring	10	209	1.1	10	196	0.9	155
		Sprat	14	301	1.8	14	255	1.6	183
		Stickleback	7	120	0.4	7	116	0.3	99
		Total	31	451	3.3	31	401	2.8	302
	Larvae	Sprat	8	102	0.6	8	39	0.02	–
*COI*	Adult	Herring	10	408	1.1	10	404	1.1	378
		Sprat	13	440	1.1	13	439	1.1	404
		Stickleback	8	96	0.6	8	93	0.6	89
		Total	31	645	2.9	31	638	2.8	589
	Larvae	Sprat	9	437	0.9	7	40	0.06	–

**Figure 1:**
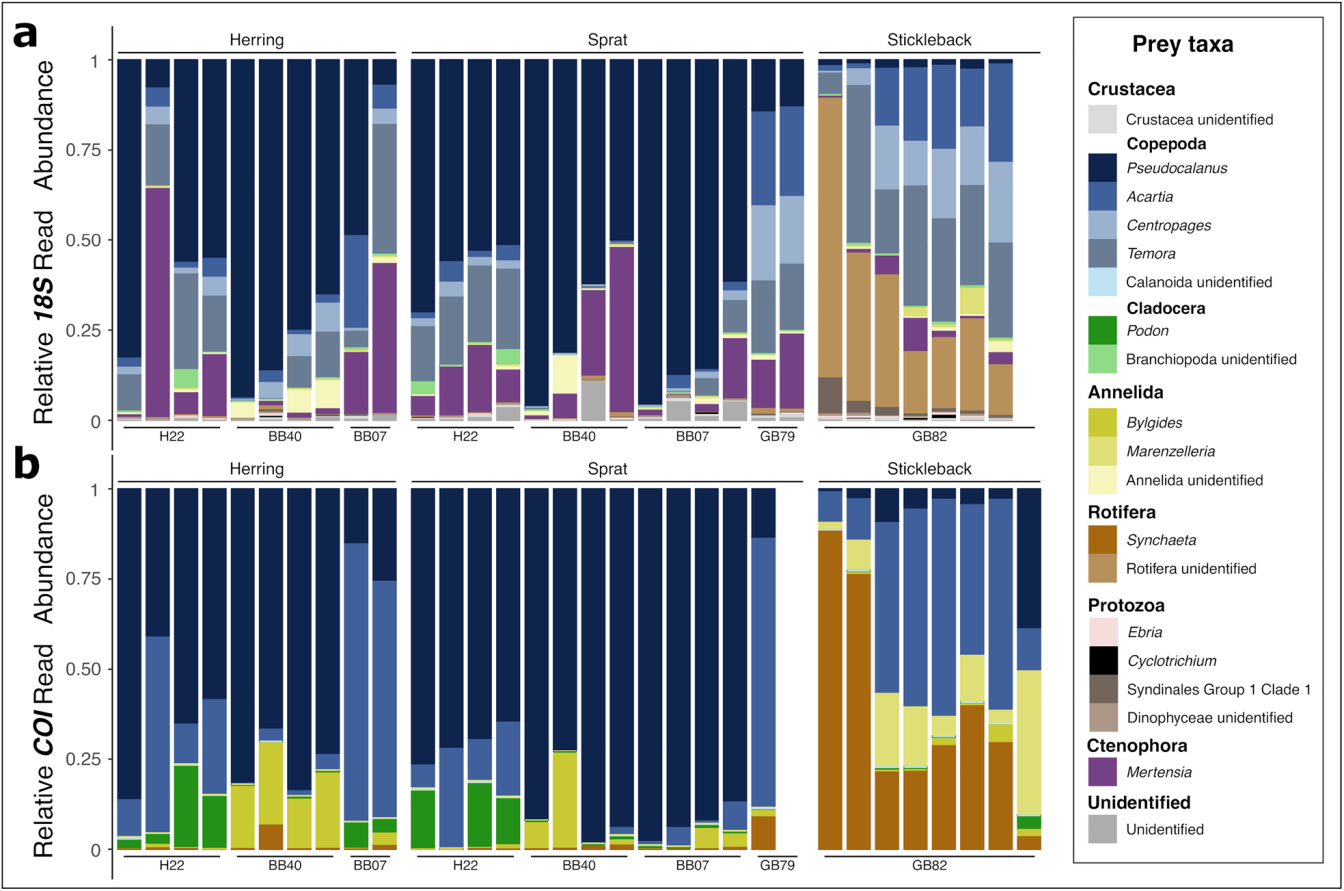
Prey composition of the planktivorous fish species herring (*Clupea harrengus*), sprat (*Sprattus sprattus*) and stickleback (*Gasterosteus aculeatus*) in the southern Baltic Sea shown as relative abundance of (**a**) *18S rRNA* and (**b**) *COI* sequence read per prey taxa. The bars represent unique biological replicates.

The relative abundances of *18S* and *COI* reads revealed overall consistent prey composition for all fish life stages, however, the taxonomic resolution differed among the barcodes (Fig. 1 and Supplementary Fig. S1). More specifically, for adult fish, *18S* had a higher resolution of copepod prey, revealing the consumption of the genera *Pseudocalanus*, *Acartia*, *Centropages* and *Temora*, whereas *COI* was limited to the detection of *Pseudocalanus* and *Acartia* (Fig. 1). Additionally, *18S* amplified *Mertensia* prey that were not amplified by *COI*. Contrarily, *COI* was better able to identify rotifers at the species level revealing the consumption of *Synchaeta baltica* and this barcode also detected cladocerans (e.g., *Podon*) and annelids, including *Bylgides* and *Marenzellaria*, at the genus level. These taxonomic differences resulted in an overall higher alpha diversity of *18S* than *COI* (Supplementary Fig. S2).

**Figure 2:**
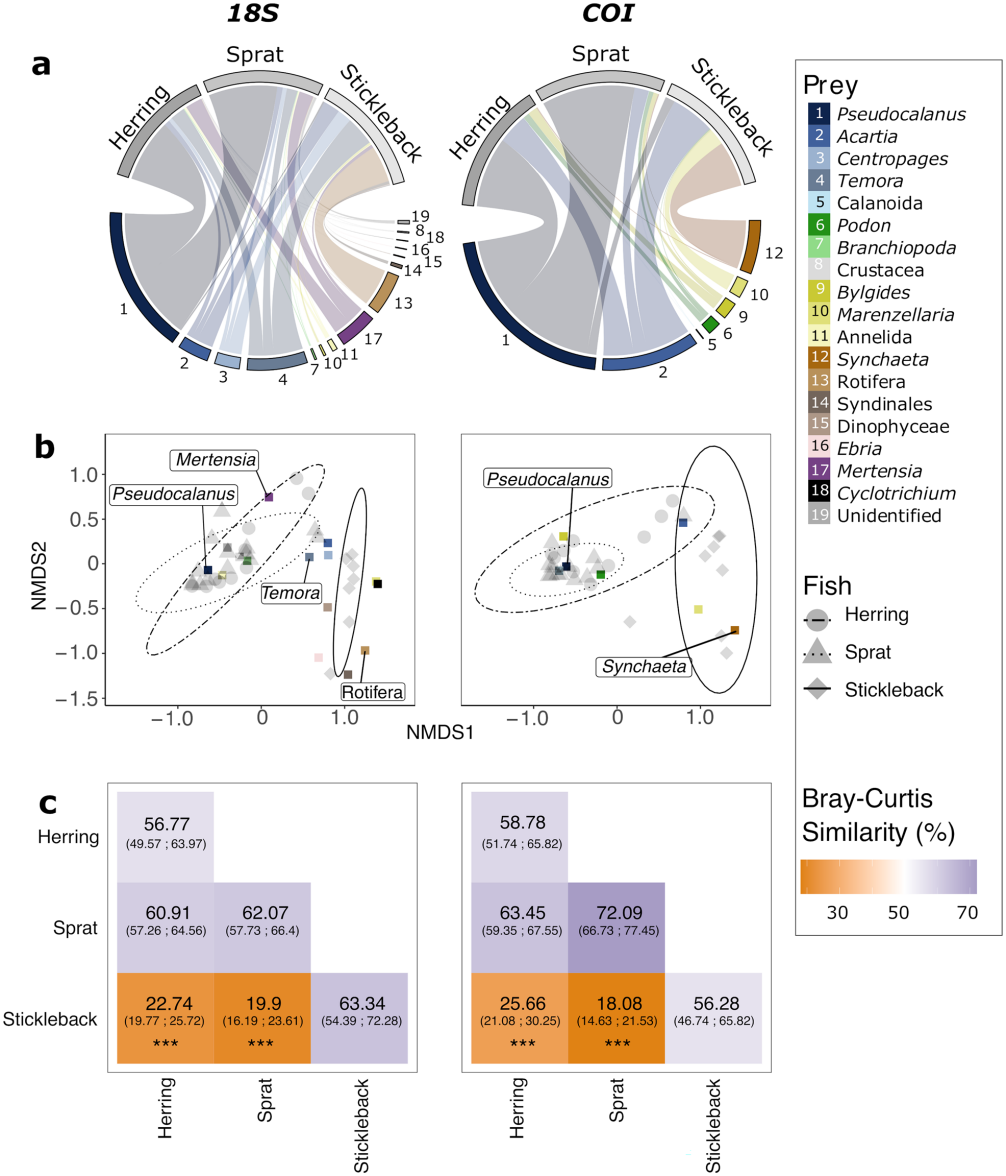
(**a**) Prevalent prey (lower) of the planktivorous fish herring (*Clupea harrengus*), sprat (*Sprattus sprattus*) and stickleback (*Gasterosteus aculeatus*) (upper) based on their gut-content analysis using DNA sequences in the southern Baltic Sea. The thickness of the links is proportional to the relative *18S rRNA* (left) and *COI* (right) read abundance. (**b**) Nonparametric multidimensional scaling (NMDS) plot of fish prey composition based on Bray–Curtis distance of *18S rRNA* (left) and *COI* (right) gut content sequences. The prey species are represented with the coloured squares and the prey contributing to the most difference between fish species is shown with their names. Ellipses following the t-distribution of the NMDS scores are shown for each fish species. (**c**) Bray–Curtis similarity Index (BCI) in percent with the 95% confidence interval in the bracket between and across (diagonal) each fish species. A low BCI indicates low niche overlap. The output of the pairwise permANOVA is shown with the asterisks (*** = *P* < 0.001).

The sequences of adult fish gut contents were dominated by several copepod species, reaching almost three quarters of their diet (73% for *18S* and 74% for *COI*), followed by ctenophores (11%, *18S*) and rotifers (11% for *18S* and 14% for *COI*) (Fig. 2a). Together, these prey taxa were responsible for at least 70% of the Bray–Curtis dissimilarity between all adult fish species (Fig. 2b). The copepod *Pseudocalanus* occupied a higher proportion of sequence reads in the gut content of herring (*18S*: 57%; *COI*: 60%) and sprat (*18S*: 63%; *COI*: 79%) compared to stickleback (*18S*: 1.5%; *COI*: 8%; *P* < 0.001, Supplementary Table S1). Based on the *18S*, the copepods *Temora, Centropages,* and *Acartia* were abundant in the sequence reads of all fish species. However, the relative abundance of *Temora* reads were twice as high in stickleback (27%) compared to herring (13%, *P* = 0.038) and sprat (10%, *P* = 0.0064, Supplementary Table S1). The ctenophore *Mertensia* was detected in higher proportions in the sequence reads of herring (15%) and sprat (13%) compared to stickleback (3%), but the beta regression revealed no significant differences between the fish species (*P* > 0.05, Supplementary Table S1). The *18S* showed that rotifers constituted about a third of stickleback’s gut content sequence reads (34%), while being almost absent in the gut content of herring (0.2%) and sprat (0.4%) (*P* < 0.001, Supplementary Table S1). More specifically, the *COI* revealed that the rotifer *S. baltica* was more associated with stickleback (39%) than with sprat and herring (ca. 1%, *P* < 0.001, Supplementary Table S1). Based on the Bray–Curtis distance of both gene markers reads, the diet of the two clupeids sprat and herring overlapped at more than 60% (Fig. 2c), which was confirmed by pairwise permANOVAs (Supplementary Table S2). Stickleback occupied a different niche than sprat (BCI_*18S*_ = 19.9%, BCI_*COI*_ = 18.08%, *P* < 0.001) and herring (BCI_*18S*_ = 22.74%, BCI_*COI*_ = 25.66%, *P* < 0.001). Moreover, ANOVA based on the Shannon Index revealed that stickleback fed on a higher diversity of prey than the two clupeids (F_2,58_ = 10.016, *P* < 0.001, Supplementary Fig. S2). Few rotifer reads were associated to sprat larvae (0.04% and 8.7% for *18S* and *COI*, respectively). However, both barcodes showed a strong contribution of copepods and cnidarians to sprat larvae diet, while *18S* identified about three times more taxa than *COI* (Supplementary Fig. S2).

**Figure 3:**
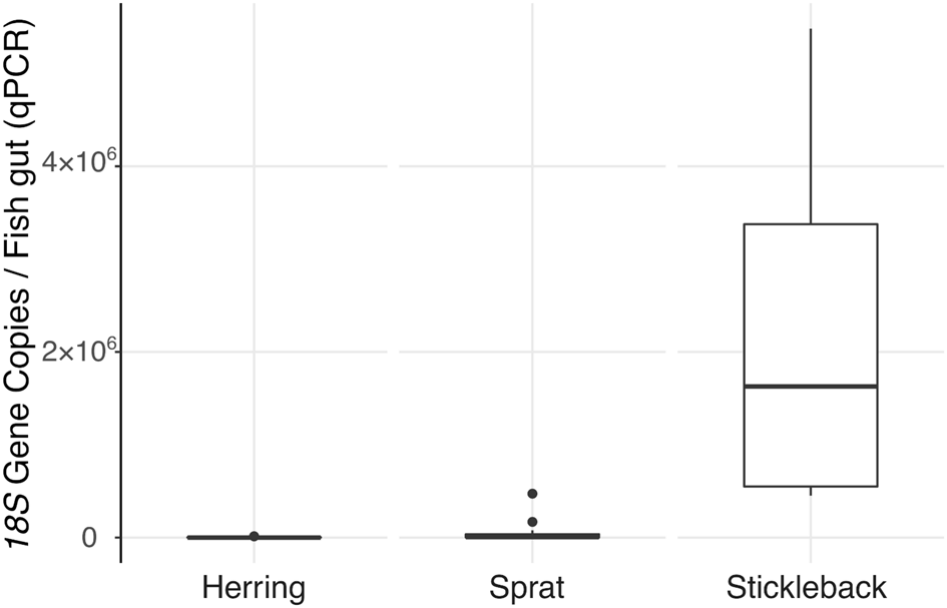
Quantitative PCR assay of rotifers in the gut content of the planktivorous fish sprat, herring and stickleback in the southern Baltic Sea targeted by rotifer-specific *18S rRNA* gene primers. The results are shown as equivalents to rotifers *18S* gene copies.

Results from qPCR verified high abundances of rotifers in the gut content of stickleback (on average, a signal equivalent to > 2.29 × 10^6^ rotifers *18S* gene copies per stickleback gut) (Fig. 3). This concentration was more than 40 times as high in stickleback as in herring or sprat (ANOVA of the log-transformed concentrations, F_2,26_ = 8.45, *P* = 0.0015). The qPCR found no detectable concentration of rotifer *18S* associated to the copepods *Acartia*, *Temora*, *Centropages*, *Eurytemora*, and *Pseudocalanus*, nor to the cladocerans *Evadne* and *Bosmina* (data not shown).

**Figure 4:**
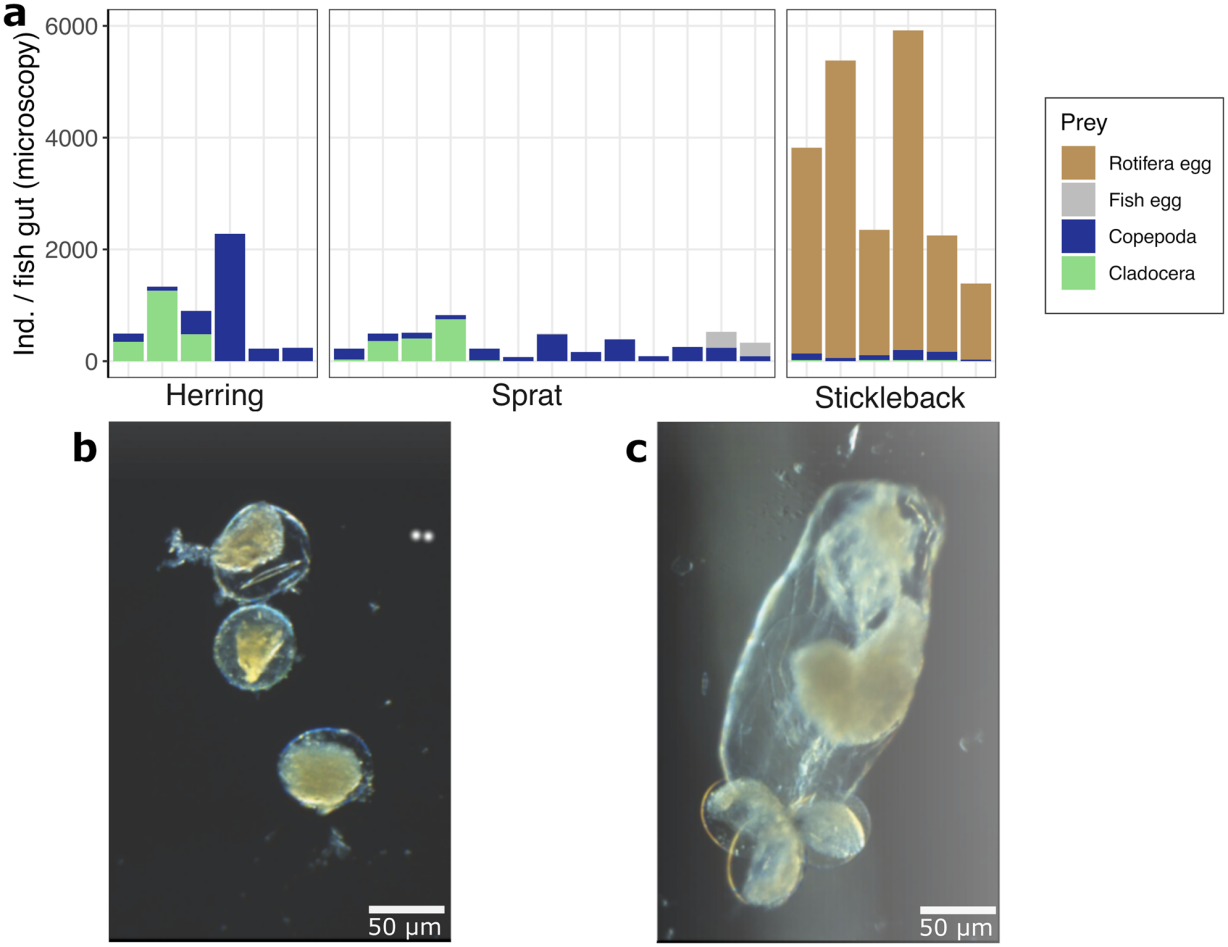
(**a**) Microscopic count of gut content of the planktivorous fish. Rotifers were identified as rotifer eggs. (**b**) Eggs were observed in the gut content of stickleback, originating from the rotifer genus *Synchaeta*. (**c**) Female of the rotifer genus *Synchaeta* carrying three eggs as identified from water samples. Scales are the same for (**b**) and (**c**).

The microscopic analysis of gut content revealed that copepods were more abundant in sprat (on average 192 copepods per gut, representing 54% of their prey) and herring (565 copepods, 62%) compared to stickleback (105 copepods, 3%) (Fig. 4a). We also found cladocerans to be abundant in some of the clupeid’s guts (on average 120 cladocerans per gut in sprat, 34% of their prey and 347 in herring guts, 38% of their prey) but less abundant in the stickleback guts (13 cladocerans per gut, 0.4%). Stickleback guts were mainly filled with rotifer eggs and contained on average 3400 eggs per gut, while no rotifer eggs were detected in sprat nor herring (Fig. 4b,c).

## Discussion

High diet overlap is assumed to cause competition between the three dominant pelagic planktivorous mesopredators in the Baltic Sea, sprat, herring, and stickleback^11,24,25^. Despite this assumption, stickleback populations have increased dramatically over the past decades, which raises the question of whether and how resources are partitioned^26^. While previous studies of fish diet overlap have mainly relied on microscopic identification of gut content, we implemented a DNA metabarcoding approach targeting two different gene regions, the *18S rRNA* gene (*18S*) and the mitochondrial cytochrome *c* oxidase I gene (*COI*) to reveal the taxonomic diversity of prey, and a qPCR step to quantify rotifers that are at times abundant in the Baltic Sea. Our study highlights consistency between methods, with DNA metabarcoding resolving the plankton-fish link at the highest taxonomic resolution. Our results suggest a unique niche of stickleback that may enable high population growth in the open water, despite high competition between mesopredators, although this finding needs to be confirmed at larger scale. More than half of the DNA found in herring and sprat stomach contents was assigned to *Pseudocalanus*, supporting previous observations of high diet overlap between the two clupeids^11,12^. On the other hand, the diet of stickleback differed substantially from the two clupeids, with rotifers appearing as main prey DNA in spring. The high rotifer biomass in the environment and lack of competition from other predators indicate that this novel niche utilization may support the drastic increase of pelagic stickleback in the Baltic Sea.

We find that copepods dominated the gut content of the two clupeids sprat and herring. *Pseudocalanus* and *Temora* occupied most of the sequence reads of the clupeid metabarcoding, two species that are often reported as preferred prey in previous studies^11,12^. Despite high contributions of these two copepods, *Pseudocalanus* was more than four times as abundant as *Temora* in clupeid gut contents. A strong preference for this copepod with marine origin can further confirm the increased competition between the clupeids, as *Pseudocalanus* has decreased due to decreased salinity^12^ and shares a similar vertical distribution as clupeid during daytime^27^. Our study using metabarcoding further reveals a large relative quantity (11%) of the ctenophore *Mertensia* in the gut samples of both clupeids. Similar, Clarke et al.^28^ reported an important contribution of gelatinous zooplankton to upper trophic levels in the Southern Ocean. Despite high abundances of ctenophores in the Baltic Sea and their assumed importance in marine food webs^19^, they are not reported as food for planktivorous fish. A possible explanation is the difficulty observing them microscopically, as their digestion rate is faster than crustaceans^29^, and no hard parts remain in the digestive system. Further, *COI* detected the presence of cladocerans, which was confirmed by the microscopic survey, but underrepresented with *18S* that strongly amplify copepods^20^. Interestingly, more than twice annelid *COI* reads, including the benthic macroinvertebrates *Bylgides* and *Marenzellaria*, were associated to stickleback (15%) and herring (8%) than to sprat (4%), highlighting their ability to migrate vertically. These interactions suggest that together stickleback and herring contribute to benthic-pelagic coupling when oxygen is not restricting vertical migration in the southern Baltic Sea^30^.

Sprat and herring share a similar feeding niche, which may explain previously observed declines in body mass and stomach fullness, and supports the theory of competition between the two species^31^. In contrast, stickleback revealed little diet overlap with the other mesopredators. The low relative abundances of *Pseudocalanus* (1–8%) in metabarcoding analyses indicates that the density-dependent competition may not limit the population growth of stickleback. The copepods that were shared in the diet of stickleback, sprat, and herring were *Temora*, *Acartia,* and *Centropages* have increased over the last decades, as opposed to *Pseudocalanus*^32^. Our results show that stickleback are able to feed on a broader spectrum of prey and highlight that stickleback utilizes the rotifer *Synchaeta baltica* as prey, which is an important component of the plankton community composition in the Baltic Sea^18,20^. Due to the difference of prey size, we can expect an overrepresentation of copepod to rotifer sequences compared with microscopic count data. High predation rate on *S. baltica* is supported by both the qPCR assay as well as microscopic counts, although only the eggshells were visible but not the soft-bodied rotifer. Despite the considerably lower carbon content per *S. baltica* (ca. 6 µg C ind^−1^) compared to copepods (ca. 20 µg C ind^−1^)^33^, the high number of rotifers likely act as a major food source for stickleback. These results propose that stickleback, due to their opportunistic feeding behaviour^34^ and smaller size^35^, have a distinct feeding niche from sprat and herring in the open water, as they feed on a smaller size class of zooplankton compared to the clupeids. Thus, we cannot assume the same process of competition between clupeids and stickleback.

Rotifers can at times be very abundant in the Baltic Sea, reaching densities up to 25,000 ind m^−3^, but their natural predators are poorly studied. An increasing trend in biomass of the two main rotifer genera (*Synchaeta* and *Keratella*) was observed since the 1990s^36^. In a recent study, we showed that rotifers might occupy a unique feeding niche, as direct grazers of dinoflagellate spring bloom, as well as in the recycling of organic matter in summer^20^. The low level of predation on rotifers by clupeid adults (< 1% of the reads) observed here indicate that this trophic niche may not be fully utilized. Further, qPCR did not identify predation on rotifers by other zooplankton species, including several species of copepods and cladocerans, which is supported by previous observations showing that limited DNA reads were associated to dominant zooplankton species in spring^20,22^. Thus, stickleback appears to have little or no competition for rotifers as a food resource in the southern Baltic Sea in spring. This abundant and increasing resource^36^ may therefore sustain the expanding Baltic stickleback population during its pelagic phase.

Similar feeding patterns but different taxonomic resolutions were found between approaches for adult fish. DNA metabarcoding has the highest taxonomy resolution, targeting the full prey spectrum, which allow for an exploration of the whole prey community for fish and other organisms^37,38^. As previously reported by Clarke et al.^39^, both barcodes showed consistent outcomes despite their different taxonomy resolutions. *18S* identified the highest prey diversity by having an increased resolution for copepods genera revealing the consumption of *Pseudocalanus*, *Acartia*, *Centropages*, and *Temora*, while *COI* was limited to the identification of the genera *Pseudocalanus* and *Acartia*. Moreover, *18S* identified gelatinous zooplankton prey, while *COI* was more efficient in identifying cladocerans, annelids and rotifers at lower taxonomy levels. qPCR, that is more sensitive than DNA metabarcoding for identifying a single species^40^, allowed us to confirm and quantify the interaction between the rotifer *S. baltica* and stickleback. However, this approach does not allow for a diet assessment, but rather for a post-hoc quantification, due to the specific primers used. Microscopy is the traditional method to assess fish diet, but is limited to prey with hard remaining parts, such as exoskeleton^23,41^. Our visual observations are consistent with the fish prey species identified at the same sampling location in 2020 (J. Hentati-Sundberg, pers. comm.) and both show the above-mentioned limitation. Our study confirms that combining molecular tools, including DNA metabarcoding and qPCR, with traditional microscopy observations is a robust approach to explore the full prey spectrum of planktivorous fish, and reveals a diverse prey spectrum for mesopredators in the Baltic Sea.

In this study, we highlight the importance of soft-bodied zooplankton organisms as potential prey for planktivorous fish, suggesting that these neglected prey taxa might be important players in the ecosystem. While several prey species are difficult to observe using traditional gut content analysis, molecular techniques such as metabarcoding and qPCR can be used as a suitable complement. In this study, we show that rotifers, which are often not identified with microscopic analysis of gut samples, may contribute to niche partitioning between sprat, herring, and stickleback. Although this result must be confirmed by more intensive sampling, the opportunistic feeding in the pelagic Baltic Sea may reduce competition with clupeids and support the increasing stickleback population.

## Methods

### Sampling, DNA extraction and microscopic analysis

**Figure 5:**
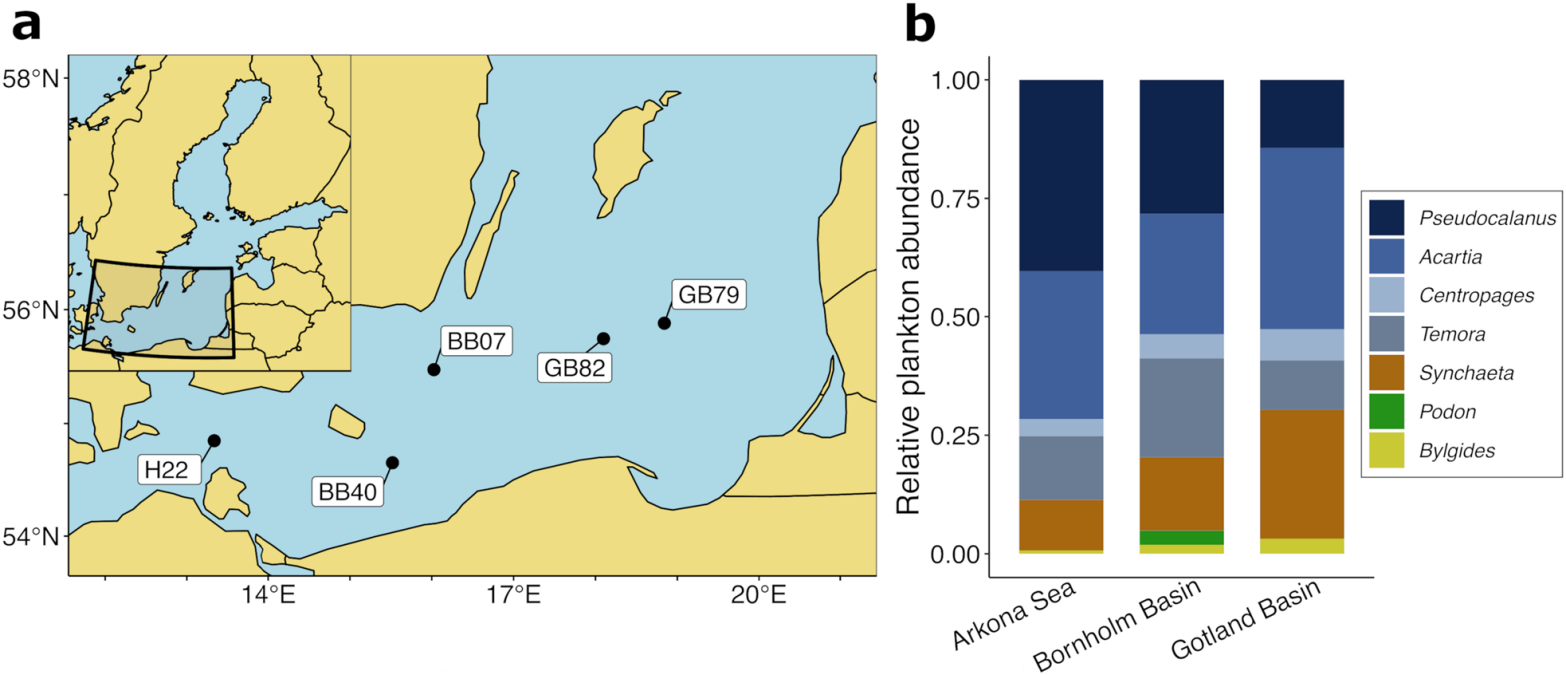
(**a**) Sampling stations in the southern Baltic Sea. H22 is located in the Arkona Basin, BB07 and BB40 in the Bornholm Basin, and GB79 and GB82 in the Gotland Basin. (**b**) Community composition (abundance from microscopy counts) of zooplankton in April 2019 at the three sampling basins. Data was downloaded from the Swedish database for pelagic monitoring^42^.

Adult and larval fish were sampled during cruise AL521 with Research Vessel ALKOR at five locations in three basins of the Southern Baltic Sea proper in April 2019 (Fig. 5a, Supplementary Table S3). Based on the database for Swedish national monitoring data^42^, potential fish prey (i.e., zooplankton) abundance in the upper 60 m (43 m in the Arkona Basin) of the water column in April 2019 was dominated by copepods, including *Pseudocalanus*, *Acartia*, *Temora* and *Centropages* contributing to more than 69% of the mesozooplankton community across all basins, and the rotifer *Synchaeta* that was more than twice as abundant in the Gotland Basin (27%) than in the Arkona Sea (11%, Fig. 5b). Adults of the three dominant pelagic planktivorous fish species, the three-spined stickleback (*Gasterosteus aculeatus*), Atlantic herring (*Clupea harengus*), and European sprat (*Sprattus sprattus*), were sampled using a pelagic fishing trawl net (“Jungfischtrawl”, 0.5 cm mesh size in the cod) deployed at specific depths during daytime between 47 and 73 m depth (Supplementary Table S3). Full stomachs of individual close to the average population size were immediately dissected, preserved in 80% ethanol and frozen at – 20 °C until further analysis. Planktonic larvae of sprat were sampled using a Bongo Net (300 and 500 µm, Hydrobios, Kiel, Germany) with single-oblique tows to 6 m above ground and back to the surface, identified and sorted under a stereomicroscope, preserved in 80% ethanol and frozen at – 20 °C until further analysis. Fish larvae were soaked in a 1% bleach solution to remove contamination of external DNA, rinsed multiple times in DNA-free water (Qiagen), and soaked in 180 µl ALT lysis buffer. The content of the fish guts was removed and homogenized, half of the content was mixed with 360 µl ALT lysis buffer (Qiagen, Hilden, Germany), and the other half was preserved in 80% ethanol for visual observation. DNA from all samples was extracted using QIAamp DNA Micro Kit (Qiagen) according to manufacturer’s instructions for tissue samples with an additional step of bead beating with 1 mm glass beads and an overnight incubation at 56 °C with proteinase K (Qiagen). We also included archived DNA samples from rotifer, copepod, and cladoceran species sampled in the northern Baltic Sea proper in 2017 and 2018 (for details see Zamora-Terol et al.^22^) to quantify rotifer to zooplankton prey contribution. The gut content of a subsample, including six herrings, 13 sprats and six sticklebacks (Supplementary Table S3), was identified under a stereomicroscope to validate the findings of the DNA metabarcoding.

### Metabarcoding analysis

A 400 bp long fragment of the V4 region of the *18S rRNA* gene (*18S*) was amplified in a polymerase chain reaction (PCR) with universal primers 528F and 706R^43^ and a 313 bp long fragment of the mitochondrial cytochrome *c* oxidase I gene (*COI*) was PCR amplified with versatile primers mlCOIintF and dgHCO2198^44^. The thermocycler conditions are described in Zamora-Terol et al.^22^ for the *18S*. For the *COI*, the thermocycler was programmed as follow: denaturation (98 °C) for 1 min, followed by 30 cycles of denaturation (98 °C) for 20 s, annealing (46 °C) for 20 s, and elongation (72 °C) for 15 s. The program finished with a final elongation (72 °C) for 2 min and a cooling (4 °C). Illumina sequencing library preparation was performed according to best practices described by Hu et al.^45^. Sequence clustering was done “onboard” and sequenced on MiSeq (MSC 2.5.0.5/RTA 1.18.54) pair-end setup (2 × 300 bp, version 3, Illumina, San Diego, California) with the addition of 10% genomic PhiX. FastQ files were processed in the cutadapt software^46^ to remove primers and adapters. The next steps of quality control, including trimming, filtering, and taxonomy assignment were performed in R using the DADA2 pipeline^47^. *18S* sequences were assigned to the Protist Ribosomal Reference database^48^. *COI* sequences were assigned to a custom-made database combining the MARES database^49^ with a 636 bp length sequence of *Synchaeta baltica* retrieved in GenBank (accession: MK905848^50^). Library preparation, sequencing, and subsequent bioinformatic analyses were performed as described earlier^20^. For each step in the library preparation a negative control was included, and analysed with Qubit and gel electrophoresis after the full library preparation. No detectable bands or DNA concentrations were observed in the negative controls.

### Quantitative PCR assessment of rotifers

To accurately assess rotifer abundance in fish and zooplankton guts, we designed a quantitative PCR (qPCR) assay by constructing a degenerate rotifer-specific primer pair to target a 94 bp long fraction of the V4 region of *18S*: Rotifer18SF (AYCGGTTGGCYGTTDATG) and Rotifer18SR (CAGGCRTAWRGCCTGCTTTA). The primers were analysed in the Test Prime software of (arb-SILVA) and match with 59.4% of the species entries of the rotifer order Monogononta, including *Keratella* spp. and *Synchaeta* spp., the two most dominant rotifer genera in the Baltic Sea. The primers did not match any other sequence in the SILVA database^51^. The primers' self-dimerization and hairpin formation was tested by “Multiple Primer Analyzer” software (Thermo Fisher) with no positive detection. The qPCR assay was performed on all fish gut samples (Supplementary Table S3) and DNA samples from copepods and cladocerans sampled previously^22^.

Each PCR reaction contained 10 µl SYBR Green I master mix (Roche, Basel, Switzerland), 1 µl Rotifer18SF and 1 µl Rotifer18SR (10 nM, Eurofins Genomics, Ebersberg, Germany), 6 µl PCR grade water (Roche) and 2 µl DNA template. qPCR was performed on LightCycler® 480 II (Roche) with an initial denaturation and polymerase activation at 95 °C for 5 min followed by 40 cycles of 95 °C for 30 s denaturation, 64 °C for 30 s annealing and 74 °C for 30 s elongation and fluorescent acquisition. All analyses were terminated by a melting curve analysis of 95 °C denaturation for 1 min followed by a gradual increase of 2.2 °C s^−1^ from 65 to 97 °C under constant acquisition. For each qPCR plate we included a standard curve to correct for primer efficiency bias, and a negative control to detect contamination. Standard curves were made from PCR products extracted and amplified DNA of *S. baltica.* Residuals of the PCR reaction was removed with GeneJET PCR purification kit (Thermo Fisher, Waltham, Massachusetts), quantified with Qubit dsDNA HS assay and fluorometer (Thermo Fisher), and subsequently diluted with a factor of 10X, starting at an absolute concentration of 10^8^ gene copies reaction^−1^ (2 µl). The standard curve was used for absolute sample copy number estimation in the Light Cycler 454 software (Roche), with Cq values defined as the highest value of the second derivative of the RT amplification curve. While amplification bias was measured and corrected for in each run, the average runs had an efficiency of 1.82 (91%) with an R^2^ of 0.993.

### Data analysis

Data filtering and statistical analysis were facilitated by the Phyloseq R package^52^. *18S* sequences belonging to the Teleostei family and *COI* sequences belonging to the sampled fish genus were removed. Subsampling using the function “rarefy_even_depth” from the Phyloseq R package^52^, based on the sample with the lowest reads count (8437 (*18S*) and 20,334 (*COI*)) were performed to homogenize the prey diversity among adult fish samples. This step was not performed for larval stages as the number of reads were too low in some samples. Different cut-offs between larvae and adult fish were used to keep dominant prey reads. For fish larvae, unidentified sequences and taxa occupying less than 5% of the reads as well as samples with less than 50 reads after filtration were removed. For adult fish, the most prevalent prey, determined as taxa occupying at least 0.1% of the sequences in at least 70% of the samples in each sample group (i.e., fish species) were kept for further analyses.

For adult fish, we used the Bray–Curtis Index ((1 – Bray–Curtis distance) × 100) to measure the percentage of dietary overlap. Differences in diet composition were tested by permANOVA using the “adonis” function from the Vegan R package^53^, and pairwise comparisons between fish species were performed using the “pairwise.adonis2” function from the pairwiseAdonis R package^54^. Prey taxa contributing to at least 70% of the difference between all fish species were assessed using the “simper” function from the Vegan R package^53^, and their relative proportions were modelled with beta regression, using the “betareg” function from the betareg R package^55^. Nonmetric multidimensional scaling plots were based on Bray–Curtis distances and scores were calculated with the “metaMDS” function in the Vegan R package^53^. Figures were made in the ggplot2 R package^56^. The most important prevalent taxa were visualized in bipartite networks made using the Circlize R package^57^.

### Ethics statement

The study protocol was approved on the 22nd of January 2019 by the Department of Ecology Environment and Plant Sciences (DEEP), Stockholm University, Stockholm, in collaboration with the GEOMAR Helmholtz Centre for Ocean Research Kiel, Kiel, in compliance with the German animal protection laws “TierSchG §4” and “TierSchlV §1 (2)”. The German animal protection laws do not require a permit for the mass capture and killing of fish from trawl net hauls and fish larvae from Bongo net tows. We confirm that the study was undertaken with all procedures that minimize the pain and suffering, and improve animal welfare.

## Supplementary Information

Supplementary Information.

## Data Availability

Raw DNA sequences generated during the current study were uploaded with associated metadata to the European Nucleotide Archive (ENA) under the project accession number PRJEB51972 (https://www.ebi.ac.uk/ena/browser/view/PRJEB51972). R scripts and data analyzed for this study are available in the Dryad repository, 10.5061/dryad.vq83bk3vk. For data request, please contact the corresponding author Kinlan M.G. Jan.
