# Supplementary material for: Niche partitioning between planktivorous fish in the pelagic Baltic Sea assessed by DNA metabarcoding, qPCR and microscopy
Source: Sci Rep. 2022 Jun 29;12:10952. doi: 10.1038/s41598-022-15116-7 (PMC9242992; doi:10.1038/s41598-022-15116-7)
Supplement: Supplementary file 1 — Supplementary Information. [file 41598_2022_15116_MOESM1_ESM.pdf]

Niche partitioning between planktivorous fish in the pelagic Baltic Sea assessed by DNA metabarcoding, qPCR and microscopy

Supplementary material

Andreas Novotny[1,3,4], Kinlan Mehdi Goulwen Jan[1,4,*], Jan Dierking[2], and Monika Winder[1]

[1]Department of Ecology, Environment, and Plant Sciences, Stockholm University, Stockholm, Sweden.

[2]GEOMAR, Helmholtz Centre for Ocean Research Kiel, Kiel, Germany.

[3]Present address: Institute for the Oceans and Fisheries, The University of British Columbia, Vancouver, Canada.

[4]These authors contributed equally: Andreas Novotny and Kinlan Mehdi Goulwen Jan

[*]email: kinlan.jan@su.se

Content

Supplementary figures

Supplementary Figure S1. ____ 3

Supplementary Figure S2. ____ 4

Supplementary Figure S3. ____ 5

Supplementary tables

Supplementary Table S1. ____ 6

Supplementary Table S2. ____ 7

Supplementary Table S3. ____ 7

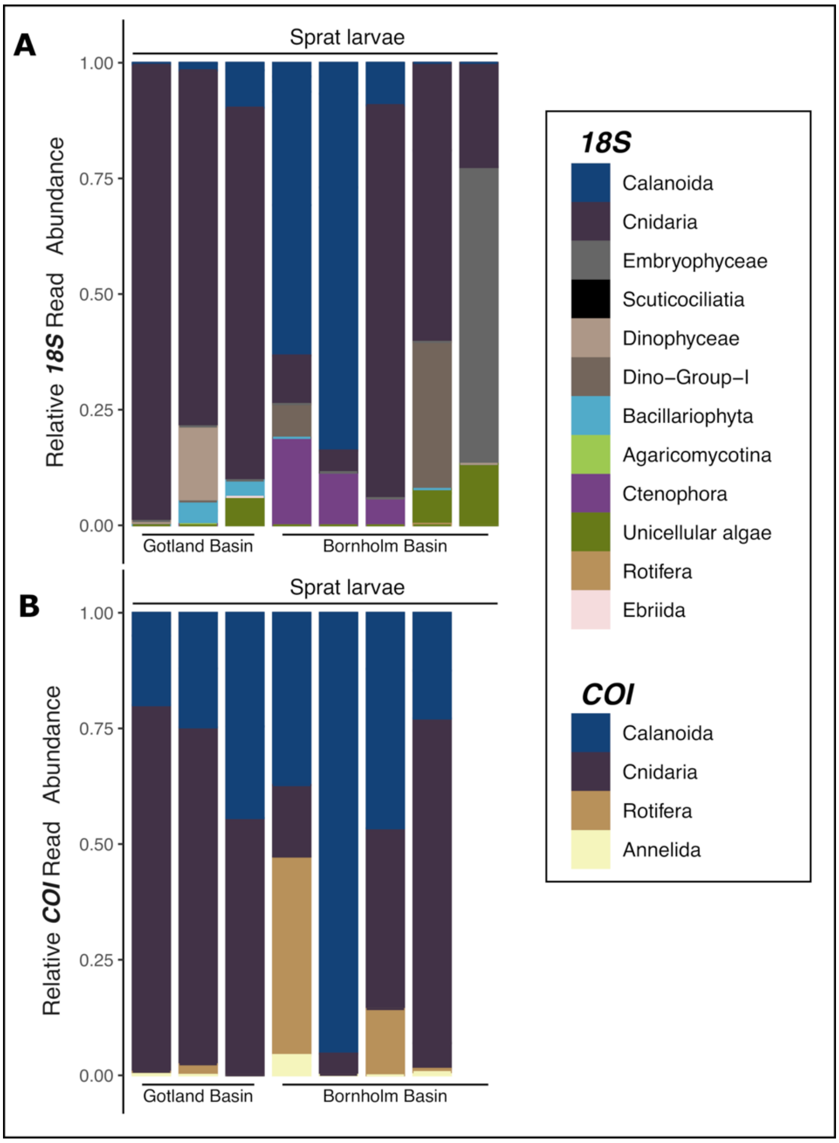

Supplementary Figure S1. Prey composition of the larval fish species sprat (*Sprattus sprattus*) shown as relative abundance of A) *18S rRNA* and B) *COI* sequence count per prey taxa. The bars represent unique biological replicates.

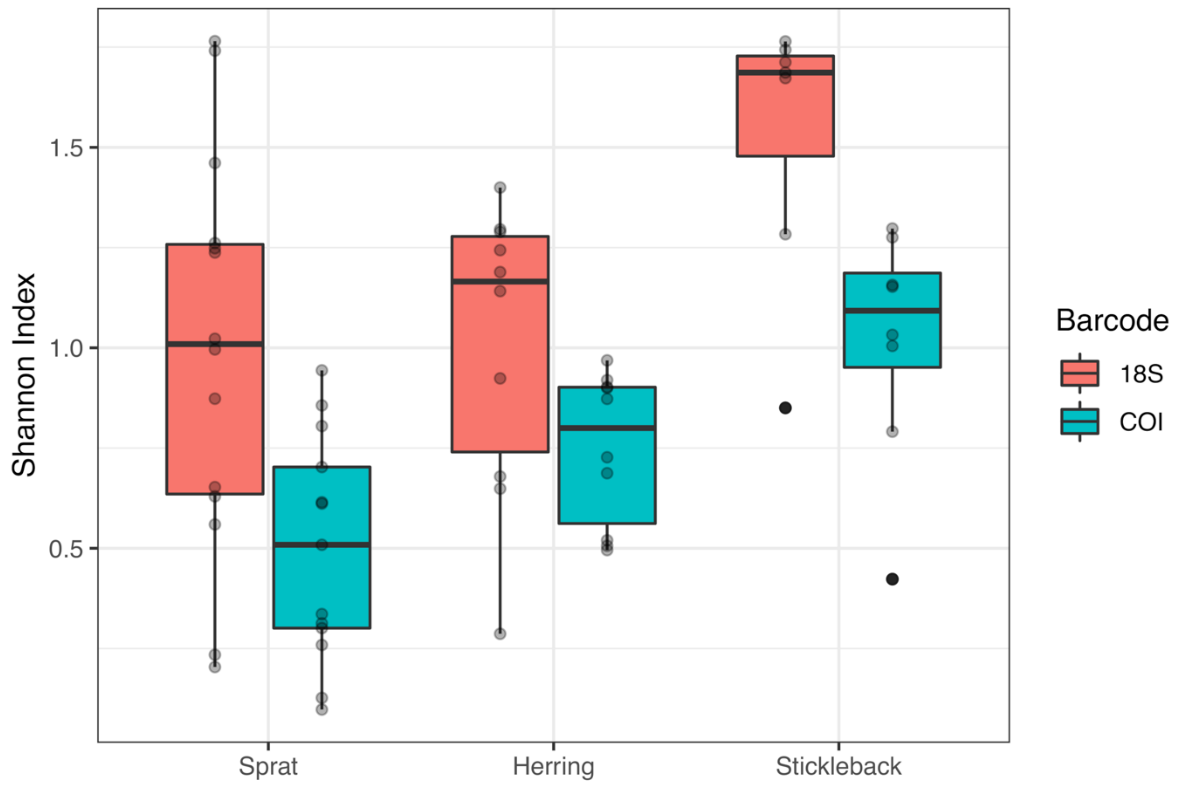

Supplementary Figure S2. Alpha diversity (Shannon Index) of prey communities for all fish species based on the *18S* and *COI* barcodes.

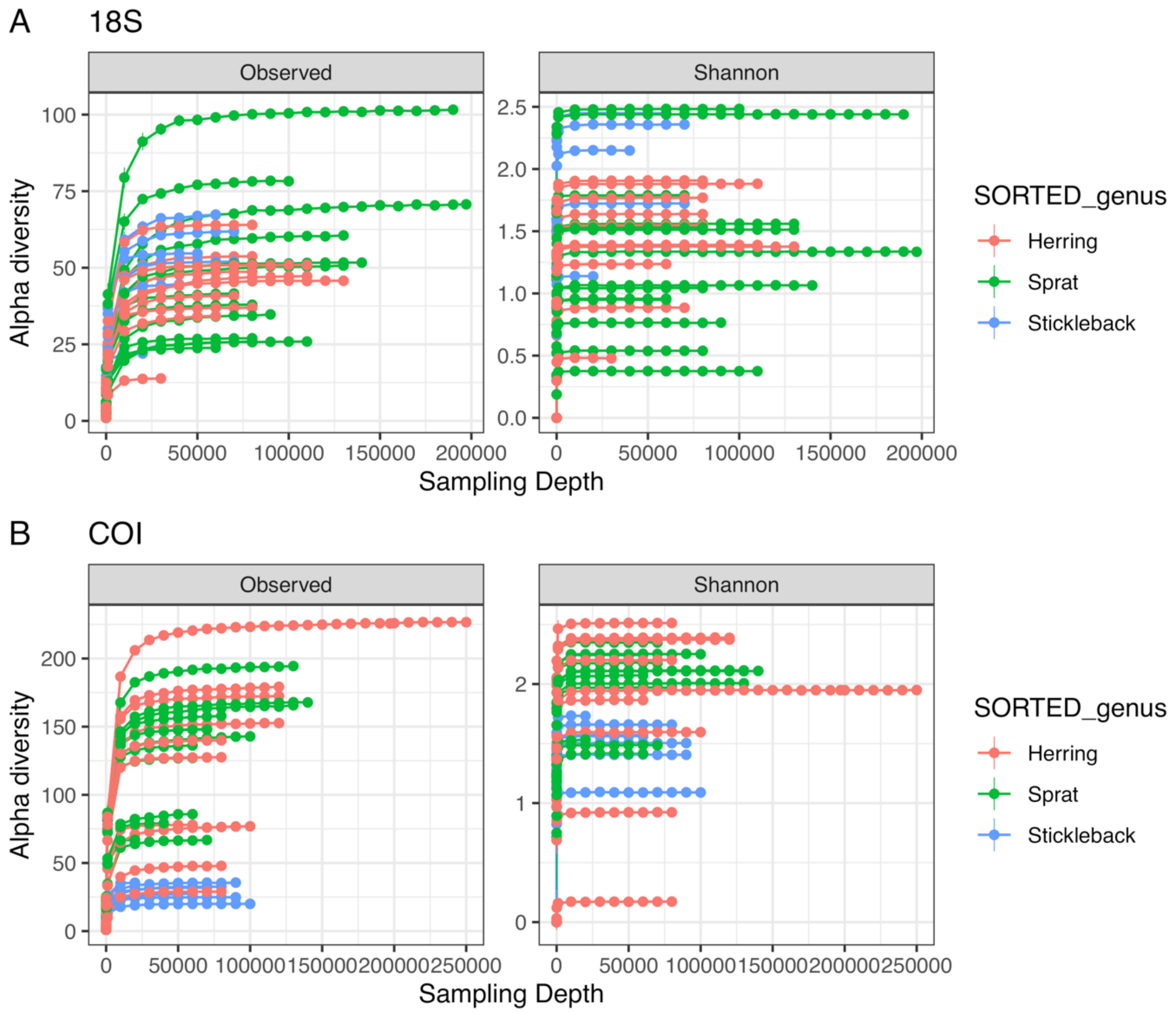

Supplementary Figure S3. Rarefaction curves showing the observed alpha diversity (left) and Shannon index (right) for *18S* (A) and *COI* (B).

Supplementary Table S1. Beta regression output for the prey contributing to more than 70% of the Bray-Curtis distance between all fish species. P values < 0.05 are shown in bold.

**Gene region**	**Prey**	**Pairs**	**Estimate**	**SE**	**Z ratio**	**P value**
18S	*Pseudocalanus*	Herring – Sprat	-0.067	0.1	-0.667	0.7824
		Herring – Stickleback	0.453	0.09	5.121	**$9.08\ 10^{-7}$**
		Sprat – Stickleback	0.519	0.08	6.724	**$5.32\ 10^{-11}$**
	Rotifers	Herring – Sprat	-0.004	0.01	-0.416	0.909
		Herring – Stickleback	-0.314	0.05	-6.311	**$8.32\ 10^{-10}$**
		Sprat – Stickleback	-0.31	0.05	-6.231	**$1.39\ 10^{-9}$**
	Temora	Herring – Sprat	0.028	0.04	0.785	0.713
		Herring – Stickleback	-0.161	0.07	-2.454	**0.038**
		Sprat – Stickleback	-0.189	0.06	-3.056	**0.0064**
	Mertensia	Herring – Sprat	-0.027	0.04	-0.616	0.812
		Herring – Stickleback	0.029	0.04	0.673	0.779
		Sprat – Stickleback	0.056	0.04	1.309	0.39
COI	*Pseudocalanus*	Herring – Sprat	-0.193	0.08	-2.334	0.051
		Herring – Stickleback	0.464	0.08	5.722	**$3.16\ 10^{-8}$**
		Sprat – Stickleback	0.657	0.07	9.862	**$2.84\ 10^{-14}$**
	Synchaeta	Herring – Sprat	$5.1\ 10^{-5}$	0.02	0.002	0.999
		Herring – Stickleback	-0.341	0.07	-4.753	**$5.97\ 10^{-6}$**
		Sprat – Stickleback	-0.341	0.07	-4.769	**$5.54\ 10^{-6}$**

Supplementary Table S2. Pairwise permANOVAs of Bray-Curtis distances among fish gut content based on the *18S* and *COI* gene regions.

**Gene region**	**Pairs**	**df**	**F**	**R^2**	**P values**
18S	Sprat -Herring	1;22	0.225	0.010	0.822
	Sprat – Stickleback	1;19	26.455	0.582	**0.001**
	Herring - Stickleback	1;15	16.502	0.524	**0.001**
COI	Sprat -Herring	1;21	1.754	0.077	0.187
	Sprat – Stickleback	1;19	29.853	0.611	**0.001**
	Herring - Stickleback	1;16	14.551	0.476	**0.001**

Supplementary Table S3. Fish trawl sampling information, including site, date and time, depth and sample size (n) for each fish species (herring, sprat, and stickleback) and methodological approach (metabarcoding, qPCR, and microscopy).

				**Metabarcoding (n)**			**qPCR (n)**			**Microscopy (n)**		
**STATION ID Basin name**	**Latitude Longitude**	**Date Start-Stop**	**Sampling depth (m)**	Herring	Sprat	Stickleback	Herring	Sprat	Stickleback	Herring	Sprat	Stickleback
BB07 Bornholm Basin	55°37.10’N 16°00.15’E	2019-04-25 13:34-14:00	73	2	4	0	2	4	0	1	3	0
BB40 Bornholm Basin	54°47.91’N 15°29.76’E	2019-04-28 10:06-10:28	74	4	4	0	3	4	0	3	4	0
GB79 Gotland Basin	55°58.87’N 18°59.50’E	2019-04-22 13:07-13:45	75	0	2	0	0	4	0	0	2	0
GB82 Gotland Basin	55°52.46’N 18°10.92’E	2019-04-22 06:18-06:48	63	0	0	7	0	0	4	0	0	6
H22 Arkona Basin	54°56.01’N 13°14.78’E	2019-04-18 14:05-14:35	47	4	4	0	4	4	0	2	4	0
